# Molecular characterization and antibiotic resistance profile of ESBL-producing *Escherichia coli* isolated from healthy cow raw milk in smallholder dairy farms in Bangladesh

**DOI:** 10.14202/vetworld.2023.1333-1339

**Published:** 2023-06-13

**Authors:** Azimun Nahar, A. K. M. Azharul Islam, Md. Nazimul Islam, Mohammad Kamruzzaman Khan, Md. Shahed Khan, A. K. M. Anisur Rahman, Md. Mahbub Alam

**Affiliations:** 1 Department of Medicine, Bangladesh Agricultural University, Mymensingh 2202, Bangladesh; 2 Department of livestock services, Ministry of Fisheries and Livestock, Krishi Khamar Sarak, Farmgate, Dhaka 1215, Bangladesh; 3 Department of Community Medicine, Mymensingh Medical College, Mymensingh 2206, Bangladesh

**Keywords:** ampicillin, antibiogram, *bla*CTX-M, cefotaxime, multiplex PCR, multidrug resistance

## Abstract

**Background and Aim:**

The emergence of antimicrobial-resistant bacteria, such as *Escherichia coli* in milk, is a serious public health concern as milk is considered a complete food and an important part of daily human diet worldwide, including in Bangladesh. However, there have been no reports on the molecular characterization and antibiotic resistance profile of extended-spectrum beta-lactamase (ESBL)-producing *E. coli* from milk of healthy cows in Bangladesh. Therefore, this study aimed to detect and characterize ESBL-producing *E. coli* (ESBL-Ec) in milk samples from healthy cows in smallholder dairy farms in Mymensingh district, Bangladesh, and assess the potential risk of consuming this milk.

**Materials and Methods:**

A total of 100 milk samples were collected from apparently healthy cows on smallholder dairy farms. *Escherichia coli* was isolated from the collected samples using standard methods. The detection of ESBL-Ec was performed phenotypically using cultural methods and genotypically by ESBL genetic determinants using multiplex polymerase chain reaction. Antimicrobial susceptibility testing of the ESBL-Ec isolates was performed using the disk diffusion method with 15 common antimicrobials.

**Results:**

In this study, out of the 100 samples tested, 70 (70%) were found to be positive for *E. coli*. Among these, 41 (58.6%) strains were identified as ESBL-producing, both phenotypically and genotypically, with the presence of *bla*CTX-M, *bla*TEM, and *bla*SHV individually or combined (*bla*CTX-M plus *bla*TEM plus *bla*SHV). The antibiogram of these ESBL-positive isolates revealed high resistance against commonly used antibiotics, such as ampicillin, cefotaxime, and gentamicin (100%), azithromycin (88%), oxytetracycline (27%), nalidixic acid, cotrimoxazole/trimethoprim (24%), and streptomycin (22%). In addition, one isolate showed resistance to 4^th^ generation of cephalosporin (cefepime). Most importantly, extensive multidrug resistance was found in many ESBL-Ec isolates. However, the isolates were highly sensitive to drugs such as ceftriaxone (100%) and imipenem (100%). This is the first study to detect ESBL-Ec in raw milk from healthy cows on smallholder dairy farms in Bangladesh.

**Conclusion:**

More than 58% of the *E. coli* isolated from raw milk of healthy cows tested positive for ESBL production and showed resistance to most commonly used antimicrobials which may be alarming for human health. A limitation of our study is that we had a small size of sample collected from one district in Bangladesh. Therefore, a larger sample size covering a wider geographic area, and using multi-locus sequence typing and whole genome sequencing could provide a more comprehensive understanding of the prevalence and characteristics of ESBL-Ec in Bangladesh.

## Introduction

*Escherichia coli* harboring extended-spectrum beta-lactamase (ESBL) gene has been considered as a global threat to human health in the past decade [1, 2]. The extensive application of antibiotics in human medicine, veterinary medicine and agriculture results in antibiotic-resistant bacteria, including ESBL-producing *E. coli* (ESBL-Ec) [1]. Extended-spectrum beta-lactamase-producing *E. coli* is widely distributed in food and wild animals, hospital settings, humans, environment and food supply chain, etc. [1]. There are several groups of ESBLs, including Temoneria (TEM)), sulfhydryl variable (SHV), and cefotaxime (CTX)-M (CTX confers resistance to cephalosporin). Temoneria and SHV were most commonly found in hospital infections in the 1980s–1990s while CTX-M became dominant with the use of the third generation of cephalosporins [3]. Globally, CTX-M is the most common ESBLs and there are five subgroups of the *bla*CTX-M gene based on amino acid sequence homology (*bla*CTX-M-1, *bla*CTX-M-2, *bla*CTX-M-8, *bla*CTX-M-9, and *bla*CTX-M-25) [4]. The *bla*CTX-M-15 member of *bla*CTX-M-1 group is the most dominant globally, including Bangladesh [5, 6]. In Bangladesh, *bla*CTX-M-1 and *bla*CTX-M-2 are prevalent [2, 7, 8]. Cefotaxime-M genes are often found on plasmids, which can also carry other antimicrobial-resistant (AMR) genes related to aminoglycosides, chloramphenicol, sulfonamide, tetracycline, and macrolides [4, 9]. In Bangladesh, dairy animals are commonly treated with penicillin, tetracycline, sulfonamides, and aminoglycosides, which may contribute to the prevalence of AMR genes in *Enterobacteriaceae* [10]. In addition, macrolides (azithromycin [AZM]) are often used to treat gastrointestinal diseases in both humans and animals in Bangladesh, as they can achieve sufficient concentration in the blood with repeated doses at 24 h intervals. Milk is an important source of macro and micronutrients, especially for infants. However, improper processing of milk and inadequate animal management can cause bacteria to multiply rapidly because milk contains high levels of nutrients [11]. Among *Enterobacteriaceae* bacteria, enterohemorrhagic *E. coli* strains can cause infections through milk, posing a significant health risk to humans [12]. The prevalence of ESBL-Ec is increasing globally, including in Bangladesh [2, 6, 13]. These pathogens are resistant to many commonly used antibiotics, creating a challenge for the treatment of infections caused by them and leading to increased use of last-resort antibiotics such as carbapenems [14]. Hence, it is important to investigate the presence of ESBL-Ec in the food processing chain or in the food we consume daily, which may come from healthy farm animals. In Bangladesh, smallholder farming systems with herd size <4 per household are commonly practiced, as they offer high economic returns with less investment compared to other cattle management systems [15].

The smallholder farming system contributes greatly to our national economy. However, farmers of smallholder dairy farms lack awareness about the rational use of antimicrobials, leading to frequent irrational use of antimicrobials as therapeutics or prophylaxis for dairy cows. Although there are a few reports on the prevalence of ESBL-Ec in drinking water [2], retail chickens [7], and milk from cows with clinical mastitis [16] in Bangladesh, no study has been conducted on the detection of ESBL-Ec in raw milk from healthy cows in smallholder dairy farms of Bangladesh and the characteristics of the ESBL-Ec in terms of ESBL genotypes and AMR pattern.

Therefore, this study was conducted to detect and characterize ESBL-Ec from healthy cow raw milk samples from smallholder dairy farms in Mymensingh district in Bangladesh.

## Materials and Methods

### Ethical approval and Informed consent

Ethical approval was not required for this study because animals were only subjected to milk collection. However, written informed consent was obtained from the owners for the participation of their animals in this study.

### Study period and location

The study was conducted from April 2021 to January 2022. A total of 100 raw milk samples were collected from healthy cows from smallholder dairy farms of 4 Upazilas (Mymensingh Sadar, Muktagacha, Phulpur, Tarakanda) in Mymensingh district.

### Sample collection

Approximately, 15 mL of milk was collected in sterile plastic containers directly from the cow’s teat. The samples were transported to the laboratory of Department of Medicine, Faculty of Veterinary Science, Bangladesh Agricultural University while maintaining a cold chain within 5–6 h. All samples were tested on the same day they were received in the laboratory. The cows were healthy and did not show any signs of mastitis. Before collecting milk samples, the udder was thoroughly cleaned and wiped with a clean, dry towel, and each teat was disinfected using 70% alcohol.

### Isolation of *E. coli*

The milk samples were enriched using a modified protocol based on a previously described method [17]. Specifically, 9 mL of sterile nutrient broth was added to 1 mL of each milk sample, and the mixture was incubated statically at 37°C for 16 h. Subsequently, a loopful of enriched culture was streaked onto MacConkey agar (HiMedia, Maharashtra, India) that contained 1 mg/L CTX (Nihon Becton Dickinson, Osaka, Japan) [18]. The dark, pink and dry *E. coli* like colonies were collected from each sample.

### Biochemical identification

All the isolates were subjected to biochemical tests such as triple sugar iron, lysine indol motility, Simmon’s citrate test, and Voges–Proskauer test to identify *E. coli* [17].

### Extended-spectrum beta-lactamase phenotyping

The ESBL production of *E. coli* isolates was determined using the double-disk synergy test. The test involved using CTX and ceftazidime (CAZ) with or without clavulanic acid (CA), as recommended by the Clinical and Laboratory Standards Institute (CLSI) [19]. An isolate was identified as an ESBL-producer if there was a 5 mm or greater increase in the zone of inhibition with CTX or CAZ disk with CA compared to CTX or CAZ alone.

### Extended-spectrum beta-lactamase gene grouping

Bacterial DNA was extracted by boiling 1 mL of overnight culture, as described by Parvin *et al*. [7]. Extended-spectrum beta-lactamase gene grouping (*bla*TEM, *bla*SHV, *bla*CTX-M-1, and *bla*CTX-M-2) was performed by multiplex polymerase chain reaction (PCR) using primer set and PCR conditions [7]. In brief, amplification reactions were set in a 25-μL volume containing 12.5 μL of PCR master mix (New England Biolabs, Massachusetts, USA), 1.0 μL (10 pmol) of each of the forward and reverse primers, 1 μL of DNA, and 3.5 μL of nuclease-free water. Polymerase chain reaction was run using T100 thermal cycler (Bio-Rad Laboratories, Inc., California, USA) with multiplex PCR conditions: initial denaturation at 95°C for 5 min, followed by 25 cycles of denaturation at 95°C for 30 s, annealing at 60°C for 1 min, and extension at 72°C for 1 min, with a final extension at 72°C for 10 min. Appropriate positive and negative controls (sterile phosphate buffer saline) were included in each PCR run. The PCR products were visualized by electrophoresis on a 1.5% agarose (TaKaRa, Shiga, Japan) gel containing ethidium bromide. The DNA bands were photographed using a UV transilluminator (Cell Biosciences, Victoria, Australia).

### Determination of antimicrobial susceptibility

The antimicrobial susceptibility of the ESBL-Ec isolates was tested by the disk diffusion method [19] using commercially available disks (Nihon Becton Dickinson, Osaka, Japan; Bio-maxima, Lublin, Lubelskie, Poland; HiMedia) against 15 antimicrobials belonging to 10 antimicrobial classes. Antimicrobial classes were considered according to CLSI guidelines [19]. They included penicillin (ampicillin [AMP, 10 μg]), cephems ([CTX, 30 μg], CAZ [30 μg], cefoxitin [FOX, 30 μg], ceftriaxone [CRO, 30 μg]), cefepime (FEP, 30 μg), carbapenem (imipenem [IPM, 10 μg]), aminoglycosides (gentamicin [GEN, 10 μg], streptomycin [STR, 300 μg]), quinolone (nalidixic acid [NAL, 30 μg]), fluoroquinolone (ciprofloxacin [CIP, 5 μg]), tetracycline (oxytetracycline [OTC, 30 μg]), phenicol (chloramphenicol [CHL, 30 μg]), macrolide (AZM, 15 μg), and sulfonamides/dihydrofolate reductase (cotrimoxazole-trimethoprim [SMZ/TMP, 25 μg]) In brief, ESBL-Ec kept as glycerol stock at −80°C were sub-cultured on nutrient agar and 3–5 *E. coli* colonies were collected and suspended in 5 mL of sterilized saline. The suspension was adjusted to achieve turbidity equivalent to 0.5 McFarland standards. An evenly distributed bacterial lawn was prepared on Mueller–Hinton agar plates using sterile cotton swabs. Antimicrobial disks were placed on each bacterial lawn. The inhibition zone of each antimicrobial agent was analyzed after 16–18 h of incubation at 37°C. Results were interpreted according to the CLSI guidelines [19]. The susceptibility test used *E. coli* strain ATCC 25922 as a control strain. Multidrug resistance (MDR) was defined as resistance to at least one antimicrobial agent from three or more antimicrobial classes [20].

## Results

### Isolation and identification of *E. coli*

*Escherichia coli* like colonies were isolated and identified from 70 out of 100 raw milk samples. The percentage of *E. coli* isolated from healthy cow raw milk in smallholder dairy farms from different upazilas was 67% in Mymensingh Sadar, 83% in Muktagacha, 75% in Phulpur, and 50% in Tarakanda (Table-1).

**Table-1:** Distribution of ESBL-producing *E. coli* isolated from healthy cow raw milk in Mymensingh District between April 2021 and January 2022.

Name of the Upazilas	Number of dairy farms covered	Number of samples studied	Number of *E. coli* isolated (%)	Number of ESBL-producing *E. coli* detected (%)
Mymensingh Sadar	27	30	20 (67)	14 (47)
Muktagacha	25	30	25 (83)	16 (53)
Phulpur	19	20	15 (75)	9 (45)
Tarakanda	17	20	10 (50)	2 (10)
Total	88	100	70 (70)	41 (58.6)

*E. coli*=*Escherichia coli*, ESBL=Extended-spectrum beta-lactamase

### Phenotypic screening for ESBL production

**Figure-1:**
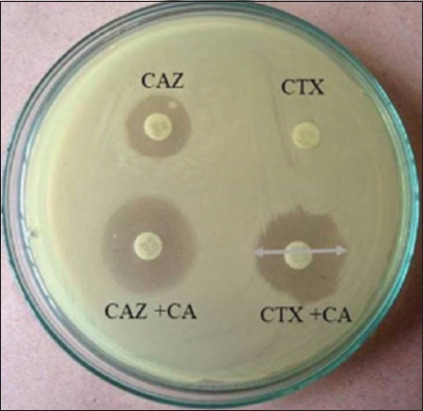
Double disk diffusion method showing extended-spectrum beta-lactamase (ESBL) production by *Escherichia coli* isolated from raw milk samples of healthy cows. The *E. coli* isolates were considered ESBL-producer when there was 5 mm or greater increase in the zone of inhibition (white arrow) with cefotaxime (CTX)/clavulanate (CA) or ceftazidime (CAZ)/CA compared to CTX or CAZ alone.

A phenotypic analysis detected an extended-spectrum beta-lactamase-producer in 41/70 *E. coli* isolates from healthy cow raw milk (Table-1). The detection was done using the double-disk diffusion method (Figure-1). Among the different Upazilas, the percentage of *E. coli* isolates from healthy cow raw milk that was ESBL producers were 47% in Mymensingh Sadar, 53% in Muktagacha, 45% in Phulpur, and 10% in Tarakanda (Table-1).

### Genotyping of ESBL-producing *E. coli*

**Figure-2:**
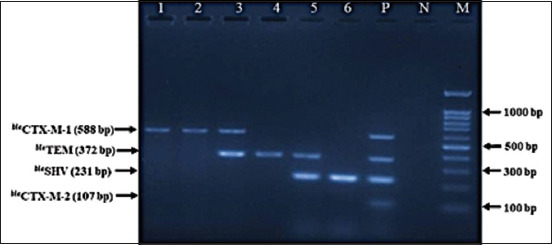
Detection of extended-spectrum beta-lactamase-encoded genes of *Escherichia coli* isolates from raw milk of healthy cow by multiplex polymerase chain reaction, separated by 1.5% agarose (TaKaRa, Japan) gel electrophoresis and ethidium bromide staining. Legends: M = 100 bp DNA ladder, Lane P = Positive control, Lane N = Negative control, Lanes 1–2=Positive for *bla*CTX-M-1 gene; Lane 3=Positive for *bla*CTX-M-1 and *bla*TEM genes; Lane 4=Positive for *bla*TEM gene; Lane 5=Positive for *bla*TEM and *bla*SHV genes; and Lane 6: Positive for *bla*SHV gene.

All of the ESBL-Ec isolates were genotypically characterized for *bla*CTX-M (*bla*CTX-M-1, *bla*CTX-M-2), *bla*TEM, and *bla*SHV. The analysis of ESBL genotype exhibited that 54% (22/41) of the ESBL-Ec carried *bla*CTX-M, followed by *bla*TEM at 24% (10/41) and *bla*SHV at 5% (2/41), whereas the combination of *bla*CTX-M with *bla*TEM was observed in 5% (2/41) and *bla*TEM with *bla*SHV in 12% (5/41) (Table-2). The study detected that *bla*CTX-M-1 was detected in 54% of ESBL-Ec isolates, but no *bla*CTX-M-2 was found. Figure-2 shows the results of the multiplex PCR assay to detect ESBL-encoding genes.

**Table-2:** The frequency of ESBL genotypes among ESBL-producing *E. coli* isolated from healthy cow raw milk in Mymensingh District between April 2021 and January 2022.

ESBL genotypes	Number of isolates (%)

	n = 41
*bla*CTX-M-1	22 (54)
*bla*CTX-M-1, *bla*TEM	2 (5)
*bla*TEM	10 (24)
*bla*TEM, *bla*SHV	5 (12)
*bla*SHV	2 (5)

n=Total number of ESBL-producing *E. coli* isolates, *E. coli*=*Escherichia coli*, ESBL=Extended-spectrum beta-lactamase

### Determination of antimicrobial susceptibility

All the isolates of ESBL-Ec obtained from raw milk showed resistance to AMP and CTX (Table-3). The highest levels of resistance were observed against GEN (100%), AZM (88%), OTC (27%), NAL, SMZ/TMP (24%), STR (22%), CIP, CAZ (18%), FOX (15%) and CHL, and FEP (2%). However, all the isolates were susceptible to CRO and IPM. Multidrug resistance was observed in *E. coli* carrying one ESBL group or their combination, including *bla*CTX-M-1 (99.9%), *bla*CTX-M with *bla*TEM (100%), *bla*TEM (80%), *bla*TEM with *bla*SHV (80%), and *bla*SHV (100%) (Table-4). Most importantly, extensive MDR (XDR), which is considered resistant to at least five classes of antimicrobials, was detected in *E. coli* harboring *bla*CTX-M-1 (27%), *bla*CTX-M with *bla*TEM (50%), *bla*TEM (30%), and *bla*TEM with *bla*SHV (20%) (Table-4).

**Table-3:** Antimicrobial resistance of ESBL-producing *E. coli* isolated from healthy cow raw milk in Mymensingh District between April 2021 and January 2022.

Antimicrobial agents	Number of resistant isolates (%)

	n = 41
AMP	41 (100)
CTX	41 (100)
CAZ	7 (18)
CRO	0 (0)
FOX	6 (15)
FEP	1 (2)
IPM	0 (0)
STR	9 (22)
GEN	41 (100)
CIP	7 (18)
NAL	10 (24)
OTC	11 (27)
CHL	1 (2)
AZM	36 (88)
SMZ/TMP	10 (24)

AMP=Ampicillin, CTX=Cefotaxime, CAZ=Ceftazidime, CRO=Ceftriaxone, FOX=Cefoxitin, FEP=Cefepime, IPM=Imipenem, STR=Streptomycin, GEN=Gentamicin, CIP=Ciprofloxacin, NAL=Nalidixic acid, OTC=Oxytetracycline, CHL=Chloramphenicol, AZM=Azithromycin, SMZ/TMP=Cotrimoxazole-trimethoprim. n=Total number of ESBL-producing *E. coli* isolates, *E. coli*=*Escherichia coli*, ESBL=Extended-spectrum beta-lactamase

**Table-4:** Multidrug resistance pattern in different ESBL gene groups carrying *E. coli* isolated from healthy cow raw milk in Mymensingh District between April 2021 and January 2022.

ESBLgene group	MDR pattern^a)^ (no. of isolate)	NARC^b)^
*bla*CTX-M-1 (n=22)	STR, NAL, SMZ/TMP, GEN, AMP, CTX, CIP, OTC, AZM (2)	7
	NAL, SMZ/TMP, GEN, AMP, CTX, CIP, OTC, AZM (1)	
	STR, NAL, SMZ/TMP, GEN, AMP, CTX, OTC, AZM (2)	6
	NAL, GEN, AMP, CTX, CIP, OTC, AZM (1)	
	STR, FOX, GEN, AMP, CTX, AZM (1)	4
	FOX, GEN, AMP, CTX, AZM (1)	
	GEN, AMP, CTX, AZM, FEP (1)	
	STR, GEN, AMP, CTX, AZM (1)	3
	GEN, AMP, CTX, AZM (10)	
	GEN, AMP, CTX (2)	2
*bla*CTX-M-1, *bla*TEM (n=2)	STR, NAL, SMZ/TMP, GEN, AMP, CTX, OTC, AZM (1)	6
	GEN, AMP, CTX, AZM (1)	3
*bla*TEM (n=10)	NAL, CHL, SMZ/TMP, GEN, AMP, CTX, CIP, OTC, AZM (1)	8
	NAL, SMZ/TMP, GEN, AMP, CTX, CIP, OTC, AZM (1)	7
	SMZ/TMP, GEN, AMP, CTX, OTC, AZM, FEP (1)	6
	GEN, AMP, CTX, AZM (5)	3
	STR, GEN, AMP, CTX (1)	2
	GEN, AMP, CTX (1)	
*bla*TEM, *bla*SHV (n=5)	NAL, SMZ/TMP, GEN, AMP, CTX, CAZ, CIP, OTC, AZM (1)	7
	FOX, GEN, AMP, CTX, CAZ, AZM (2)	4
	STR, GEN, AMP, CTX, CAZ, AZM (1)	3
	GEN, AMP, CTX, CAZ (1)	2
*bla*SHV (n=2)	FOX, GEN, AMP, CTX, CAZ, AZM (2)	4

a) AMP=Ampicillin, CTX=Cefotaxime, CAZ=Ceftazidime, FOX=Cefoxitin, FEP=Cefepime, STR=Streptomycin, GEN=Gentamicin, CIP=Ciprofloxacin, NAL=Nalidixic acid, OTC=Oxytetracycline, CHL=Chloramphenicol, AZM=Azithromycin, SMZ/TMP=Cotrimoxazole-trimethoprim;

b) NARC=No. of antimicrobial resistance classes according to CLSI (CLSI, 2012); n=Total number of isolates, *E. coli*=*Escherichia coli*, ESBL=Extended-spectrum beta-lactamase

## Discussion

We described the detection of ESBL-Ec in healthy cow raw milk for the first time in Bangladesh. The high prevalence of ESBL-Ec in raw milk and the multidrug-resistant nature of the isolates indicate a potential risk to human health. The consumption of raw milk should be avoided. The treatment of human illness caused by ESBL-Ec should be based on the antibiogram study findings to avoid further developing antimicrobial resistance.

Our study revealed that 70% of the raw milk carried *E. coli* isolates, which is much higher than a previous report (38.0%) in Sudan [21]. The prevalence of AMR bacteria has been reported in food animals and its products (meat, milk, and cheese) in many countries [22‒24]. These AMR bacteria may exert a risk to public health through the food chain as these bacteria can spread their resistance genes to other bacteria by horizontal transfer. Therefore, food such as milk contaminated with ESBL-Ec may contribute to the wide spread of ESBL-producing bacteria in the human community [25]. We found the presence of ESBL-Ec in 41% of the healthy cow milk samples collected from smallholder dairy farms in the Mymensingh district of Bangladesh. Similarly, other authors reported 23.53%–29.3% prevalence of ESBL-Ec in healthy cow raw milk samples [21, 26]. The distribution of the prevalence of ESBL-Ec in healthy raw milk within different Upazilas of Mymensingh district was similar except in Tarakanda. We observed a high level of ESBL-Ec contamination in the healthy cow raw milk, which might be an important concern for public and animal health. This study revealed that a high percentage of the isolates from raw milk harbored *bla*CTX-M (59%), *bla*TEM (41%), and *bla*SHV (17%). In contrast, in Bangladesh, 38.9% of *E. coli* isolated from milk of cows suffered with mastitis [16] and 100% ESBL-Ec isolated from frozen chicken [7] carried only *bla*TEM. This variation may be due to the difference in isolation protocol or sample sources. However, Batabyal *et al*. [27] reported that 54.5% ESBL-Ec from raw milk carried *bla*CTX-M. The *bla*CTX-M gene in *E. coli* is thought to be circulating among dairy cattle in Bangladesh. Among *bla*CTX-M genes, the isolates carried *bla*CTX-M-1 gene-group (54%) and a combination of this gene with *bla*TEM (5%) but no isolates harbored *bla*CTX-M-2 gene-group. According to geographical distribution, the prevalence of *bla*CTX-M-1, *bla*CTX-M-2, and *bla*CTX-M-9 genes under *bla*CTX-M group have been reported in Asia [18, 28]. Hence, a higher prevalence of *bla*CTX-M-1 in *E. coli* from raw milk origin may be relevant. Moreover, many ESBL-Ec isolates harbored the combination of *bla*CTX-M plus *bla*TEM and *bla*TEM plus *bla*SHV genes. This indicates the increasing emergence of ESBL-Ec in Bangladesh, possibly due to travelers from abroad where ESBL-Ec is prevalent.

It is well known that the plasmids harboring ESBL genes can also carry resistant genes against many other antimicrobials such as aminoglycosides, sulfonamide chloramphenicol, and tetracycline [4]. In this study, ESBL-Ec isolated from raw milk showed the highest resistance to GEN followed by AZM, OTC, SMZ/TMP, and NAL. Resistant to TET, GEN, and sulfonamides were also reported in ESBL-Ec isolated from different sources from Bangladesh [6, 7] and India [27]. It is known that GEN, TET, and sulfonamides are often used in dairy farms as therapeutics and prophylaxis in Bangladesh. It is important to note that indiscriminate or excessive use of these drugs in both human and animal healthcare settings can contribute to the development and spread of antimicrobial resistance. However, it is unclear why most of the ESBL-Ec are highly resistant to AZM. One of the reasons is that AZM has often been used against the bacteria *Enterobacteriaceae*, causing gastrointestinal diseases in humans in many countries, including Bangladesh [9], which might result in the emergence of resistance to these antibiotics in food animals through the food chain. Nowadays, beta-lactam antibiotics and macrolides are commonly used antimicrobials for treating human patients. Therefore, it is important to continuously investigate any emergence of these drug-resistant bacteria in animal-based food products for human consumption, including raw milk. We observed a high prevalence of MDR in different groups of ESBL-carrying *E. coli* isolated from raw milk. The high prevalence of MDR and XDR ESBL-Ec in this study highlights the potential for these bacteria to serve as a reservoir of resistance genes and pose a threat to public health [10].

## Conclusion

The prevalence of ESBL-Ec in healthy cow raw milk is very high and consumption of raw milk should be avoided. Most of the ESBL-Ec we detected were MDR and XDR, which can easily be transmitted to humans as consumers and, thus, result in potential health hazards. Therefore, it is important to raise awareness among the public and stakeholders about the potential risks associated with the consumption of raw milk and need to adopt appropriate measures to prevent the spread of AMR pathogens. This is the first study to report the detection of ESBL-Ec in healthy cow raw milk in Bangladesh.

## Authors’ Contributions

AN, MMA, and AKMAR: Conceptualization. AN, AKMAI, MNI, MKK, and MSK: Methodology. AN, AKMAI, MNI, MKK, and MSK: Original draft preparation. MMA and AKMAR: Editing and supervision. All authors have read, reviewed, and approved the final manuscript.

## Data Availability

The supplementary data can be available from the corresponding author on a reasonable request.

## References

[1] Ben Said L, Jouini A, Klibi A, Dziri R, Alonso C.A, Boudabous A, Ben Slama K, Torres C (2015). Detection of extended-spectrum beta-lactamase (ESBL)-producing *Enterobacteriaceae* in vegetables, soil and water of the farm environment in Tunisia. Int. J. Food Microbiol.

[2] Mahmud Z.H, Kabir M.H, Ali S, Moniruzzaman M, Imran K.M, Nafiz T.N, Islam M.S, Hussain A, Hakim S.A.I, Worth M, Ahmed D, Johnston D, Ahmed N (2020). Extended-spectrum beta-lactamase-producing *Escherichia coli* in drinking Water samples from a forcibly displaced, densely populated community setting in Bangladesh. Front. Public Health.

[3] Wei X, Wang W, Lu N, Wu L, Dong Z, Li B, Zhou X, Cheng F, Zhou K, Cheng H, Shi H, Zhang J (2022). Prevalence of multidrug-resistant CTX-M extended-spectrum beta-lactamase-producing *Escherichia coli* from different bovine faeces in China. Front. Vet. Sci.

[4] Bonnet R (2004). Growing group of extended-spectrum beta-lactamases:The CTX-M enzymes. Antimicrob. Agents Chemother.

[5] Naseer U, Sundsfjord A (2011). The CTX-M conundrum:Dissemination of plasmids and *Escherichia coli* clones. Microb. Drug Resist.

[6] Mazumder R, Abdullah A, Ahmed D, Hussain A (2020). High prevalence of blaCTX-M-15 gene among extended-spectrum β-lactamase-producing *Escherichia coli* isolates causing extraintestinal infections in Bangladesh. Antibiotics (Basel).

[7] Parvin M.S, Talukder S, Ali M.Y, Chowdhury E.H, Rahman M.T, Islam M.T (2020). Antimicrobial resistance pattern of *Escherichia coli* isolated from frozen chicken meat in Bangladesh. Pathogens.

[8] Lina T.T, Khajanchi B.K, Azmi I.J, Islam M.A, Mahmood B, Akter M, Banik A, Alim R, Navarro A, Perez G, Cravioto A, Talukder K.A (2014). Phenotypic and molecular characterization of extended-spectrum beta-lactamase-producing *Escherichia coli* in Bangladesh. PLoS One.

[9] Nguyen M.C.P, Woerther P.L, Bouvet M, Andremont A, Leclercq R, Canu A (2009). *Escherichia coli* as reservoir for macrolide resistance genes. Emerg. Infect. Dis.

[10] Rahman M.A, Rahman A.K.M.A, Islam M.A, Alam M.M (2017). Antimicrobial resistance of *Escherichia coli* isolated from milk, beef and chicken meat in Bangladesh. Bangl. J. Vet. Med.

[11] Gwandu S.H, Nonga H.E, Mdegela R.H, Katakweba A.S, Suleiman T.S, Ryoba R (2018). Assessment of raw cow milk quality in smallholder dairy farms in Pemba Island Zanzibar, Tanzania. Vet. Med. Int.

[12] Hickey C.D, Sheehan J.J, Wilkinson M.G, Auty M.A.E (2015). Growth and location of bacterial colonies within dairy foods using microscopy techniques:A review. Front. Microbiol.

[13] Devi L.S, Broor S, Rautela R.S, Grover S.S, Chakravarti A, Chattopadhyay D (2020). Increasing prevalence of *Escherichia coli* and *Klebsiella pneumoniae* producing CTX-M-type extended-spectrum beta-lactamase, carbapenemase, and NDM-1 in patients from a rural community with community acquired infections:A 3-year study. Int. J. Appl. Basic Med. Res.

[14] Kayastha K, Dhungel B, Karki S, Adhikari B, Banjara M.R, Rijal K.R, Ghimire P (2020). Extended-spectrum β-lactamase-producing *Escherichia coli* and *Klebsiella* species in pediatric patients visiting international friendship children's hospital, Kathmandu, Nepal. Infect. Dis. (Auckl).

[15] Quddus A (2018). Smallholder dairy farming in Bangladesh:A socioeconomic analysis. Bangl. J. Agric. Econ.

[16] Bag M.A.S, Khan M.S.R, Sami M.D.H, Begum F, Islam M.S, Rahman M.M, Rahman M.T, Hassan J (2021). Virulence determinants and antimicrobial resistance of *E. coli* isolated from bovine clinical mastitis in some selected dairy farms of Bangladesh. Saudi J. Biol. Sci.

[17] Ombarak R.A, Hinenoya A, Awasthi S.P, Iguchi A, Shima A, Elbagory A.R.M, Yamasaki S (2016). Prevalence and pathogenic potential of *Escherichia coli* isolates from raw milk and raw milk cheese in Egypt. Int. J. Food. Microbiol.

[18] Nahar A, Awasthi S.P, Hatanaka N, Okuno K, Hoang P.H, Hassan J, Hinenoya A, Yamasaki S (2018). Prevalence and characteristics of extended-spectrum β-lactamase-producing *Escherichia coli* in domestic and imported chicken meats in Japan. J. Vet. Med. Sci.

[19] CLSI (2018). Performance Standards for Antimicrobial Susceptibility Testing.

[20] Cantón R, Ruiz-Garbajosa P (2011). Co-resistance:An opportunity for the bacteria and resistance genes. Curr. Opin. Pharmacol.

[21] Badri A.M, Ibrahim I.T, Mohamed S.G, Garbi M.I, Kabbashi A.S, Arbab M.H (2017). Prevalence of extended-spectrum beta-lactamase (ESBL) producing *Escherichia coli* and *Klebsiella pneumoniae* isolated from raw milk. Mol. Biol.

[22] Yang F, Zhang S.D, Shang X.F, Wang X.R, Wang L, Yan Z.T, Li H.S (2018). Prevalence and characteristics of extended spectrum β-lactamase-producing *Escherichia coli* from bovine mastitis cases in China. J. Integr. Agric.

[23] Yu Z.N, Wang J, Ho H, Wang Y.T, Huang S.N, Han R.W (2020). Prevalence and antimicrobial resistance phenotypes and genotypes of *Escherichia coli* isolated from raw milk samples from mastitis cases in four regions of China. J. Glob. Antimicrob. Resist.

[24] Watson E, Jeckel S, Snow L, Stubbs R, Teale C, Wearing H, Coldham N (2012). Epidemiology of extended-spectrum beta-lactamase *E. coli* (CTX-M-15) on a commercial dairy farm. Vet. Microbiol.

[25] Lazarus B, Paterson D.L, Mollinger J.L, Rogers B.A (2015). Do human extraintestinal *Escherichia coli* infections resistant to expanded-spectrum cephalosporins originate from food-producing animals?A systematic review. Clin. Infect. Dis.

[26] Ali T, Ur Rahman S, Zhang L, Shahid M, Zhang S, Liu G, Gao J, Han B (2016). ESBL-producing *Escherichia coli* from cows suffering mastitis in China contain clinical class 1 integrons with CTX-M linked to IS CR1. Front. Microbiol.

[27] Batabyal K, Banerjee A, Pal S, Dey S, Joardar S.N, Samanta I, Isore D.P, Singh A.D (2018). Detection, characterization, and antibiogram of extended-spectrum beta-lactamase *Escherichia coli* isolated from bovine milk samples in West Bengal, India. Vet. World.

[28] Le Q.P, Ueda S, Nguyen T.N, Dao T.V, Van Hoang T.A, Tran T.T, Hirai I, Nakayama T, Kawahara R, Do T.H, Vien Q.M, Yamamoto Y (2015). Characteristics of extended-spectrum β-lactamase-producing *Escherichia coli* in retail meats and shimp at a local market in Vietnam. Foodborne Pathog. Dis.

